# Social robot for older adults with cognitive decline: a preliminary trial

**DOI:** 10.3389/frobt.2023.1213705

**Published:** 2023-11-24

**Authors:** David Figueroa, Ryuji Yamazaki, Shuichi Nishio, Elie Maalouly, Yuma Nagata, Yuto Satake, Miyae Yamakawa, Maki Suzuki, Hideki Kanemoto, Manabu Ikeda, Hiroshi Ishiguro

**Affiliations:** ^1^ Intelligent Robotics Laboratory, Department of Systems Innovation, Graduate School of Engineering Science, Osaka University, Osaka, Japan; ^2^ Symbiotic Intelligent Systems Research Center, Institute for Open and Transdisciplinary Research Initiatives, Osaka University, Osaka, Japan; ^3^ Department of Psychiatry, Graduate School of Medicine, Osaka University, Osaka, Japan; ^4^ Division of Health Sciences, Graduate School of Medicine, Osaka University, Osaka, Japan; ^5^ Department of Behavioral Neurology and Neuropsychiatry, United Graduate School of Child Development, Osaka University, Osaka, Japan

**Keywords:** social robot, robot acceptance, conversation robot, social assistive robot, human-robot interaction

## Abstract

The number of older adults living alone is rapidly increasing. Loneliness in older adults not only degrade their quality of life but also causes troubles such as heavy burden on the medical staff, especially when cognitive decline is present. Social robots could be used in several ways to reduce such problems. As a first step towards this goal, we introduced conversation robots into the homes of older adults with cognitive decline to evaluate the robot’s availability and acceptance during several months. The study involved two steps, one for evaluating the robustness of the proposed robotic system, and the second one to examine the long-term acceptance of social robots by older adults with cognitive decline living alone. Our data shows that after several weeks of human-robot interaction, the participants continued to use the robot and successfully integrated them into their lives. These results open the possibility of further research involving how sustained interaction can be achieved, as well as which factors contributed to the acceptance of the robot.

## 1 Introduction

By the end of the century, the population in Japan will decrease by more than half, declining from its 2017 population peak of 128 million to 53 million (Vollset et al., 2020). Currently, Japan has the largest percentage of older adults in the world, reaching 27.7% of its population in 2017. Consequently, life expectancy in Japan is of 84.2 years, the highest on Earth (Cabinet Office of Japan, 2018; World Health Organization, 2016). Therefore, the number of households exclusively formed by older adults living alone is projected to reach about 9 million by 2040 (National Institute of Population and Social Security Research, 2019). These facts indicate that individuals in the Japanese society have a long life span, and hint that it will extend more in future generations. Older adults tend to have insecure and avoidant attachment styles, which have been associated with detachment from society, potentially leading them to adverse health and psychosocial conditions such as poor cardiovascular function, impaired immunity, loss of cognitive abilities, accelerated cognitive decline, loneliness, increased risk of social isolation, clinical depression, risk of suicide, and even a particularly high-risk of solitary death (Murayama et al., 2011; Alcaraz et al., 2018; Spence et al., 2020). Loneliness is negatively correlated with cognitive function, increasing the risk of developing dementia (Boss et al., 2015; National Academies of Sciences, Engineering, and Medicine, 2020). This causes dementia to be one of the main causes of impairment and dependency among older adults over the world (Geldmacher and Whitehouse, 1996). Japan has the highest dementia prevalence among OECD countries, reaching 2.3% of the population in 2017, and is projected to reach 3.8% by 2037 (Fukawa, 2018). Cognitive impairment, including those leading to dementia, tends to be progressive. Hence, early detection, as well as prompt interventions, are needed as they can help affected individuals considerably by slowing the progress of the condition (Chong and Sahadevan, 2005).

To alleviate the burden placed on the striving healthcare sector, social robots are expected to play an important role and, recently, many studies have been conducted on this topic (Abdi et al., 2018; Papadopoulos et al., 2020; Koh et al., 2021). Surveys on such studies indicated that social robots are promising for assisting health and social care, especially on psychosocial care of older adults. However, at the same time, it is pointed out that multiple issues exist that make practical use of robots for care in real-world situations, such as in care facilities or at homes of older adults, a challenging task. Koh et al. (2021) surveyed 53 papers and classified such barriers. Amongst those, which include high cost and ethical concerns, technical issues were dominant, for instance, instability and unreliability of the robots and difficulty in adapting robots for individual needs. These concerns were especially raised on prototype robots with verbal interaction capabilities, but were also observed with pet robots and with commercial robots. Besides, such problems were more evident with older adults with cognitive impairment.

Based on these observations, our aim is to accomplish a small conversation robotic system that can be placed in homes of older adults with cognitive decline living alone for multiple months and achieve long-term interaction. Similar studies have rarely been attempted before; for this reason, the present study was focused on the development of a stable, operation-free social robot and a preliminary trial to assess its long-term usability and acceptability. Promoting the interest and usage of robots in long-term scenarios is one of the biggest challenges we have to solve in order to achieve effective human-robot interaction. We intend to provide some insights related to how this specific target group would coexist with social robots in the long term at home. Placing robots in each participant’s home and encouraging private use could allow the users to get used to them and foster successful usage in the long term. If older adults with cognitive impairment living alone accept and make use of the robot, the sustained interaction with it could gradually influence their behavior and encourage them to engage in conversations, keeping their minds active, or promote activities such as walking outside their homes or interacting with other people, helping to prevent physical problems and, therefore, reduce isolation and improve their quality of life. After fulfilling sustained, long-term interaction with social robots, it would be possible to improve the system and implement advanced functionalities such as an improved dialogue system, adaptation and customization for individual needs, and acquisition of daily physical and psychological information of older adults to be used by medical practitioners in order to provide an improved medical/nursing care.

Most research involving conversation robots in the long-term has been performed by allowing the use of robots for specific periods of time, on specified days, for a number of weeks. Rakhymbayeva et al. (2021) designed a long-term study to use social robots as a mediator between therapists and autistic children during therapy. Each interaction session with the robot lasted around 15 min and the experiment was planned to have a maximum of 10 robot-assisted sessions. Another long-term study by Céspedes et al. (2021) used social robots to improve patient motivation and adherence to cardiac rehabilitation. The study lasted 2.5 years overall, while each patient had 36 rehabilitation sessions with the robot, two times a week. Valentí-Soler et al. (2015) used multiple social robots with Alzheimer’s disease patients for a period of 3 months, 2 days a week, for 30–40 min each time. These previous studies used social robots in the long-term but most of the work was done with controlled sessions, having a specific time frame during specific days of the week and a therapist conducting the session, limiting the time a person could interact with the robot. Systematic reviews evaluating the use of various types of robots, not constrained only to conversation robots, used by people with dementia show that robots are rarely used without a time restriction (Yu et al., 2022; Hirt et al., 2021).

A notable exception is the study by Chen et al. (2020), where the companion robot Kabochan was used for a period of 32 weeks in order to improve neuropsychiatric symptoms and mental health for older adults with dementia in long-term care facilities. The study consisted of four phases, 8 weeks were used for establishing baselines, then the robot was introduced for 8 weeks, withdrawn for another 8 weeks, and finally reintroduced for the last 8 weeks. However, the study does not specify the number of robots used, which was the robot behavior during the interactions, or the impressions of the participants.

While there are number of studies involving robots designed for use by older adults, the amount of research targeting older adults with cognitive decline is still relatively small. This target group can have difficulties using the robots if they are not used to them. Kouroupetroglou et al. (2017) implemented a robot called MARIO which used multimodal interaction, to be used by older adults suffering from dementia. The participants had problems with the combination of verbal and visual cues, affecting the quality of the interaction. A study involving our target group, older adults suffering from cognitive decline and living alone, was performed by Gross et al. (2011) where a communication robot was implemented. The robot could successfully provide reminders and give instructions for cognitive stimulation, resulting in lower levels of stress, but it does so by using video calling and requires caregivers to manipulate the robot, which does not alleviate the problem of a lack of medical staff.

Experiments are rarely performed in the participants’ homes. In addition, the studies are usually performed with participants who either live with someone or reside in care houses. In a study by Schroeter et al. (2013), a mobile robot called CompanionAble was used as a companion and offered activities via the use of a touch screen to both older adults with memory impairments and their partners. Results show it provided an enjoyable experience, but the experiment was performed in an experimental smart home for 2 days, away from the participants’ homes.

Our trial was composed of two studies: a case study and the main study. In the case study, we tested and improved our robot system to achieve higher availability during long periods of time, decided on the physical settings of the robot to prevent damages while also allowing interaction with the participant. This case study had a duration of 6 months. After achieving a technical robustness that allowed sustained interaction with the robot, the main study started, in which more individuals participated in an effort to measure long-term acceptance and usage of conversation robots. The duration of the main study differed by each participant, ranging from 7 weeks to 20 weeks.

In the following sections, we describe the details of our robot in Section 2. Section 2.3 and 3 show the two studies of the trial with their respective results, and finally, we discuss the findings and future direction of research in this field in Section 4.

## 2 Materials and methods

The trial was done in Osaka, Japan, and was based on approval from the Ethics Committee at the Graduate School of Engineering Science, Osaka University (approval code: 31-3-4). All participants agreed to place our robot at their homes, after a detailed description of the trial, and written consent was obtained from both the participants and their families (their adult children).

Certain requirements need to be achieved in order to make sustained interaction possible. The robot should be available for interaction at any time of the day, so we decided to place it inside the participants’ homes. Therefore, the robot system should be compact and easy to place. This is especially important in Japan, where most houses are small and are not suitable for large mobile robots. Ideally, the robot should be maintenance-free, so the hardware must be robust, and the software should allow real-time monitoring of its status remotely. Remote support is needed for software updates or modifications on functionality during the study so there is no need for the physical presence of a third party. Finally, the robot usage should be simple enough to allow each user to focus on the conversation while requiring no special training to use it. Because of this, we chose a robust commercially available robot and made specific modifications in order to narrow the gap between commercially available robots’ functionalities and the specific functions required in this trial.

### 2.1 The original robot

**FIGURE 1:**
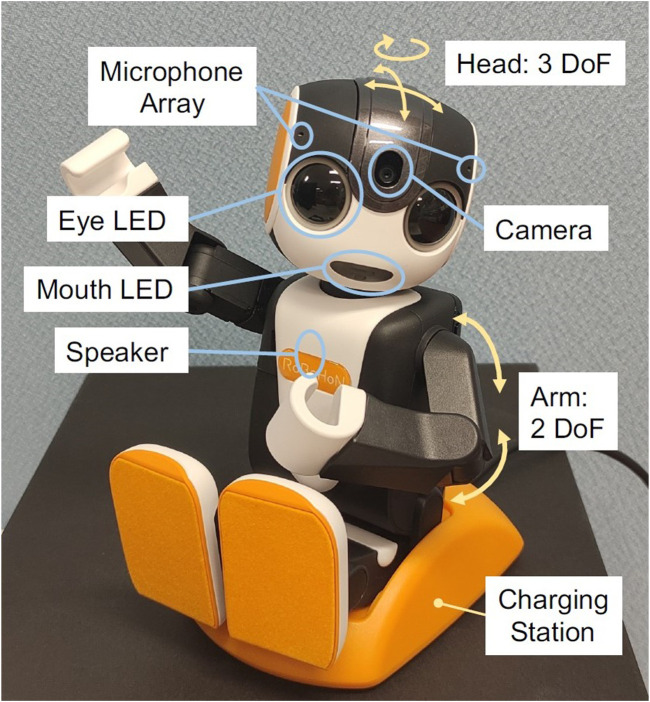
The robot sitting in its charging station. Labels show elements of the robot and degrees of freedom.

The robot chosen for the task at hand was the second-generation model of Sharp Co.’s RoBoHoN^TM^ (Sharp Corporation, 2021), which is based on the Android version 8.1 operating system, has a humanoid shape that stands 19.5 cm in height, weighs approximately 360 g, uses a Qualcomm Snapdragon 430 processor (8x ARM Cortex A53), 16 GB ROM/16 GB RAM. The device has a microphone array comprised of two microphones, which allows a rough estimation of horizontal sound source direction; a speaker, an 8-megapixel camera, a 3 axis accelerometer, a 3 axis magnetometer, a 3 axis gyroscope; and Bluetooth, Wi-Fi, and GPS capabilities. It also has LED lights placed on its mouth and eyes (Figure 1). The robot is also equipped with a touch screen on its back. The price of the robot, including its charging station and basic cloud-based speech recognition service, is approximately 100 thousand Japanese yen. This relatively low price was one of the reasons we chose this robot for the trial. More than twelve thousand units of this product has been sold in Japan (as of 2019), mostly for personal usage. We chose this robot for our trial as we expected it to be robust enough in daily use, especially in its hardware, including its actuators. This turned out to be accurate, and all the robots we used for the trial except one (described later) had no hardware issues.

The model used for the trial, SR-05M-Y, is a robot without leg actuators. This model has seven degrees of freedom (DOF) in total, two in its arms and three in its head/neck. The robot can perform speech recognition by capturing the user’s utterance, sending the audio to its speech recognition server, and receiving the recognition result. After receiving the result, the robot responds using speech and motion using a rule-based dialogue engine. These states of the robot, listening, “thinking”, and responding, are indicated by different colors and blinking speed of its eye and mouth LEDs. At the same time, the robot estimates the direction of the voice using its microphone array and moves its head toward the direction of the speech to give a natural feeling during the interaction.

The robot has three basic behaviors by default. First, it can react to voice commands related to providing information, such as weather information, and also perform predefined actions, such as singing or dancing; second, when some time has passed without having interaction, the robot can ask questions about its user, such as likes and dislikes, and learn user preferences. Lastly, after a long idle time, it can perform random actions which can induce interaction by catching the user’s attention. The robot’s voice and speech content is designed to be similar to as of a small boy. Consistently, some of the robot’s random actions display a child-like behavior, for example, when playing alone, singing, or exercising.

### 2.2 Customization for the trial

The commercial robot has been developed for hobby usage, so users are expected to have moderate technological knowledge. As our aim is to use this robot for supporting older people with cognitive decline, the robot should require no user operation. For this reason, we developed customized software in order to make robot usage simpler, focusing on voice interaction and removing unnecessary options. It is also necessary to ensure that the robot’s behavior would not change by accidental user operation, keeping its functionality persistent, while easily accepting updates if necessary. Therefore, we implemented self-monitoring and remote-control functionalities. This setup allowed us to check the robot’s status and recover from failures in the original firmware, or for any unexpected behavior triggered by an unexpected operation performed by users, allowing the research team to control the robot’s actions remotely. The customized software provided means to gather user interaction logs and send them to a remote storage system to avoid overflowing the limited local storage. These collected logs can be analyzed to improve the interaction design and experience. The system was designed to be deployed easily in less than 5 minutes; taking the robot out of the box and plugging it into a power supply are essentially all the operations required. The quick setup was essential to run the trial during the COVID-19 pandemic situation–where it is critical to not spend long periods of time in older adults’ homes.

When sound is detected, voice activity is assumed and the robot runs the automatic speech recognition (ASR) process. Based on the result of this process, the robot chooses an action to perform. When the speech is successfully recognized, the robot generates an answer to continue with the conversation. In cases where only few words are detected, the robot can either try to answer based on those words or can let the user know that it could not understand so the user can repeat the sentence. In worse case scenarios, where sound is detected but the ASR cannot detect valid speech, the robot can just reply with motion or assume it was a misdetection and ignore the voice activity.

**FIGURE 2:**
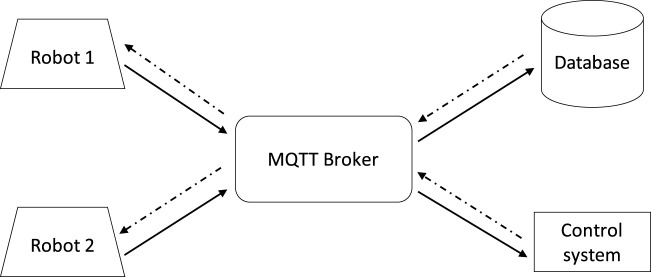
MQTT communication architecture used between the robots, and remote servers. The lightweight transportation system has flexible extendibility that allows multiple robots to be monitored and controlled by multiple servers. The system allows permanent sessions with endpoints so the communication is easy and nearly real-time. Messages can go both ways, robots can send data to the database or receive commands from the control system.

The customized software allows near real-time two-way communication using the Message Queuing Telemetry Transport (MQTT) protocol (OASIS Open, 2014). TLS was used as an underlying encryption channel to improve data security and a messaging layer was built over an MQTT layer to provide an easy exchange of multimodal data. MQTT is a publish-subscribe protocol that allows high scalability by decoupling endpoints sending a message (publisher) from those receiving it (subscriber). This is accomplished by using another component, called broker, which filters and routes incoming messages to registered subscribers. In this way, clients never interact directly with each other as all the messages pass through the broker, which also allows messages incoming from a publisher to reach multiple subscribers. This connection was chosen due to its lightweight payload, which allows fast, reliable, and relatively simple communication on limited bandwidths, while also making a persistent session possible, so robots can receive or send messages any time needed. In our system, messages sent from robots are redirected to a database for logging and, if necessary, our remote-control component can send commands to any robot to perform required actions (Figure 2).

In our trial, the basic built-in speech response functionality of the commercial robot was used while the control system relied mainly on our customized software. The robot was set to be available for interaction any time of the day, and it was programmed to perform random actions during the daytime to attract the participants’ attention and, potentially, engage in interaction. At 7 a.m. in the morning, the robot was set to give a morning greeting, emulating waking up, and signaling random actions could occur, and at 10 p.m. in the evening it would utter a good night message and stop all actions unless the participant initiated the interaction, i.e., the participant could interact with the robot, but this would not try to initiate a conversation by itself. In this way, the participants could know when the robot started looking for interaction, similar to a person’s daily active and inactive cycle. This scheduled behavior was defined in one of the remote controlling components and could be easily changed remotely if needed.

The remote functionalities can be extended to perform complex operations in future studies. For example, a conversation module can use the raw audio obtained by the robots for environmental sound detection, or use stored conversation history for advanced dialogue control. It can also be used to control dialogue flow that can be customized for individual requirements.

### 2.3 Case study

To determine details regarding the experimental environment, a reduced, case study was performed. The focus of the case study was to assess the robustness and correct problems of the equipment to allow successful interaction between robots and participants in the long term. We expected technological challenges such as software instability in continued use, as the robot should be available the whole day through months in the trial period. A challenge in the widespread use of social robots is whether the users accept the robots or not, so the case study was used to also gather initial impressions about the robot. This was an important factor before increasing the number of participants, as they may experiment a novelty effect and gradually lose interest in the robot, or might not feel comfortable with the robot’s behavior and may avoid using it. Therefore, the case study counted with only one participant living alone but with easy access to healthcare workers in case the participant needed help or, in our case, if the robot required simple manual operations such as rebooting. The conversation robot system was placed for a period of 6 months, during which the research team monitored the robot’s functionality. After this time period, unstructured interviews were performed with the participant and healthcare staff in order to investigate the robot acceptance.

#### 2.3.1 Participant details

The participant, an 88 years old female, had a Hasegawa’s Dementia Scale-Revised (HDS-R) score of 9 and was diagnosed as having Alzheimer’s disease, a type of dementia. The HDS-R is composed of nine simple questions, was initially developed in 1974 (Hasegawa, 1974) and has been widely accepted in Asian populations for clinical use and for use in epidemiological surveys for the evaluation of cognitive impairment (Imai and Hasegawa, 1994). In general terms, an HDS-R score of less than or equal to 20 corresponds to suspected dementia.

**FIGURE 3:**
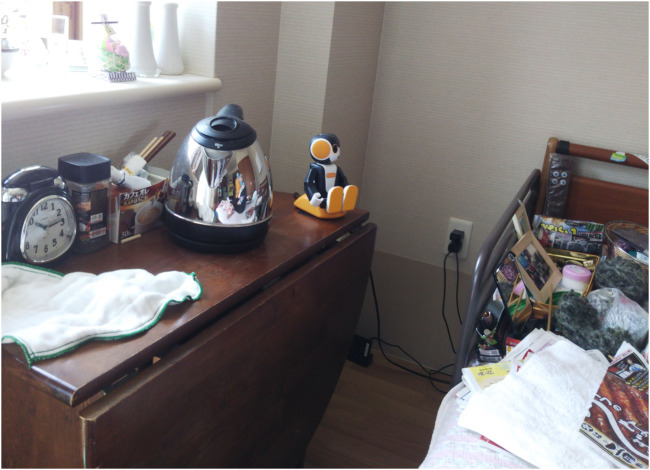
Robot setup at a participant’s home.

The participant suffered from significant memory loss, as well as episodes of disorientation, especially regarding time, and could speak only short sentences, making conversation with only one to two turns possible. The participant was able to perform daily activities but required assistance from caretakers to look after her. Therefore, the participant lived in a special kind of assisted living residence called, in English, “housing with service for older adults”, where patients reside in their own apartments but have a common shared area where healthcare workers are available. A robot, a charging station, and a mobile Wi-Fi router were placed in the participant’s home for the 6 months of duration of the case study (Figure 3). This setup allowed the participant to engage in conversation with the robot at any time. One of the reasons we asked this participant to join our trial was that in case the robot malfunctioned or caused issues, we could ask the healthcare staff to reboot the robot or to check the situation. As this trial was held during the COVID-19 pandemic period, we wanted to avoid visiting the apartment as much as possible to guarantee the safety of the participant and the ones of the researchers.

#### 2.3.2 Results

The case study allowed to evaluate the stability of the system as a whole and to make changes to the physical setup of the equipment. Both the default RoBoHoN software and the custom software needed to be stable enough to sustain long-term use. The custom software should allow, at the very least, monitor the robot status to know if there is a problem that requires the attention of an operator.

**FIGURE 4:**
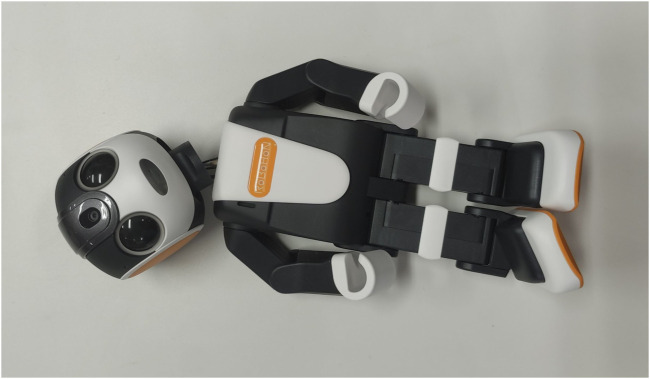
Damaged robot as a result of falling down during the first stage of the trial.

In the first month of the case study, we frequently encountered issues such as the robot’s firmware malfunction and hardware damage. Initially, the equipment placement allowed the participant to hug the robot as she wanted to interact with the robot physically. This eventually led the robot to fall and to break its neck as shown in Figure 4. Occasionally the network connection to the robot went down when the participant unplugged the mobile router. We opted for continuing the experiment by fixing the robot’s position. Therefore, the charging station position was fixed on a bedside table, and the robot was attached to it in a sitting position so it could not be moved from the established location. The mobile router was hidden behind a TV in the room. This setup was designed to avoid damages to the equipment, as well as to ensure that all the devices were always plugged to an electricity source. The use of this fixed configuration was successful in preventing damages and other problems related to physical malfunction, which is why we used this configuration for the main study of our trial too. The physical setup proved valuable when setting the robot’s environment as this problem did not repeat after fixing the robot and the router.

The research team also had to solve difficulties related to the robot’s software, which showed instability related to its continuous usage. During this study, the robot stopped its functionality on many occasions due to software malfunction. Many errors arose both from the default functions of the robot, such as out-of-memory errors from the built-in speech recognition system, as well as from the customized software. Some of these errors were due to the way the robot was used, as the original commercial robot product was designed to be used for hobbyists with some technical knowledge and not for 24/7 automatic operation. In this trial, we did not expect the users to operate the robot, but rather prevent them to change any of the robot settings. The robot itself was required to keep running for months without any intervention, if possible.

Thus, the robot needed to be configured to have high availability with no operation on the user side - in this case, by the participant with cognitive decline. Studying the data gathered and the system logs associated with unexpected events allowed the research team to successfully overcome technical issues to sustain long-time usage and develop a robust system which, in turn, allowed the robot to function appropriately for multiple weeks. The system developed during the case study allowed the robots to function as intended for the duration of the second step, the main study.

Impressions from the participant and the healthcare staff of the assisted living residence revealed that the participant kept showing interest in the robot, and after the first month, she accepted the robot into her daily routine, even creating a sense of attachment to it. The participant interacted with the robot often during the day and night, paid attention to it, and enjoyed the robot’s companionship. As the participant gradually felt more comfortable with the robot, she seemed to have started to look after it. After 3 months out of the six planned, the technical issues preventing long-term usage were solved, and the stable system configuration was used to start the main study. Instead of finishing the case study after 3 months, the robot stayed in the participant’s home for the remaining months until the mobile network contract period ended because she wanted to keep using it.

## 3 Main study

Through the first stage of the trial, the case study, we were able to stabilize the robot system and achieve continuous operation without user operation. Therefore, we extended the number of participants to study if the robot can be accepted by a larger group of older adults with cognitive decline for a long duration and evaluate if the robot has a positive effect on them. The aim of this study was to evaluate the long-term acceptance of the conversation robot by older adults with mild cognitive decline living alone. Interaction records were gathered to objectively observe changes in robot usage over time. In addition, impressions from the participants and their close relatives were collected using unstructured interviews to obtain subjective perspectives of the effect the robot could have on the participants.

The participant in the previous phase, the case study, lived in an assisted living residence where she had periodical communication with healthcare staff. In the main study, people with cognitive decline who were living alone were recruited to join the trial. As it is rare that people with dementia live alone at their home, people who joined this stage had higher cognitive ability compared to the participant in the case study, such as those diagnosed to be in the pre-dementia stage.

### 3.1 Participants

Five participants were recruited from memory clinic patients. Characteristics of participants are shown in Table 1. All the participants lived alone without any pets. Mini-Mental State Examination (MMSE) (Folstein et al., 1975) was administered to assess the overall cognitive state within 2 months before and after the study. MMSE scores of the participants ranged from 23 to 28, which indicated preserved general cognition. Clinical Dementia Rating (CDR) (Morris, 1993) was also assessed and all the participants had a CDR score of 0.5, which indicated “questionable dementia”. Three of the participants were diagnosed with pre-dementia according to the international criteria of The National Institute on Aging and the Alzheimer’s Association in 2011 (Albert et al., 2011) while two participants were diagnosed with psychosis.

**TABLE 1:** Characteristics of participants in the main study. Participant 5 had been in hospital for 2 months (58 days) during the trial.

ID	Gender	Age	Education period	CDR	Condition		MMSE		Period (days)
					Type	Duration (months)	Pre	Post	
P1	F	86	12 years	0.5	Pre-dementia	15	27	27	43
P2	F	84	9 years	0.5	Pre-dementia	216	24	25	114
P3	F	86	9 years	0.5	Psychosis	30	23	28	134
P4	F	89	12 years	0.5	Pre-dementia	48	27	28	85
P5	F	85	12 years	0.5	Psychosis	45	25	28	90 (32)

CDR: clinical dementia rating; MMSE: Mini-Mental State Examination.

### 3.2 Study settings

In each participant’s home, a robot was placed with varying duration ranging from 7 weeks to 20 weeks. The study starting date varied for each participant as they were recruited when they had a periodical medical check at the memory clinic. After they agreed to join the study, we arranged setup dates when a family member (often living in a distance) was available as well. The main study ended for all the participants at the end of the fiscal year in Japan (March 2021). Robots, as well as mobile routers, were placed at participants’ homes in a fixed location as in the case study.

In this study, as a measure to check how frequently the participants interacted with the robot, we gathered interaction logs with our MQTT-based system. The following are the details on how the robot responded to participants’ voice activities and activity recordings were performed.

1. Robot’s eye LED blinks slowly in yellow (idle state)
2. Voice activity is detected
  • Robot’s eye LED start blinking quickly in yellow
  • Audio recording starts
  • Direction of arrival of sound is obtained
3. End of voice activity is detected
  • Robot’s eye LED turns green
  • Audio recording stops
  • Robot’s head is turned to the direction of arrival of sound
  • Recorded audio is sent to the speech recognition server
4. A photograph is taken by the robot’s embedded camera
5. Speech recognition result is obtained
6. Robot’s eye LED turns orange
7. The robot responds based on the speech recognition result
8. Robot returns to idle state

Note that the robot moves its head only after the end of the voice activity is detected. This is to prevent the speech audio recording to be contaminated with gear noise. Besides, as the robot’s camera is embedded in the head, images were taken after the head motion was completed so that the voice activity source would be in the camera’s field of view. Through these steps, our system allowed us to accumulate speech recordings, speech recognition results and captured face images. Table 2 summarizes the number of voice activities detected during the trial, as well as other relevant statistics.

**TABLE 2:** Summary of voice activities detected per participant. When calculating the statistics, days without any voice activity were not included. The metrics were calculated using the filtered values. Max. and Min. value refers to the highest and lowest number of voice activities detected in a single day for each user after filtering, respectively. Q1 refers to the first quartile and Q3 to the third quartile.

ID	Total	Filtered	Average (/day)	Std. dev	Q1	Q3	Max.value	Min.value
P1	147,092	12,269	285.3	142.17	194.5	382.5	663	0
P2	58,212	9,628	84.5	64.02	34.25	109	293	3
P3	267,227	18,056	138.9	111.77	48.25	198.5	499	0
P4	253,235	11,125	132.4	63.53	90	162.75	347	25
P5	88,468	7,701	285.2	138.93	219	377.5	601	13

We also interviewed the participants and their families on their impression of the robot at the end of the study. At this point, we also asked them if there were any changes in the participants’ daily life, including their social and physical activities. The aim here was to see whether the robots were accepted by the participants as well as whether the robots had any influence on the daily life of the participants.

### 3.3 Results

In contrast to the case study, no issues with the robots were observed during the main study. However, after the trial, we found that Participant 5 (P5) had been hospitalized for 2 months (58 days) during the trial due to a mild stroke. This stroke was diagnosed to have no effect on her cognitive function. While the robot kept working, the interaction log showed no valid voice activities during those days.

#### 3.3.1 Voice activity analysis

As a measure to check how frequently the participants have been interacting with the robot, we summarized the number of voice activities detected by the robot (Table 2). While examining the data, it was found that P3 had four consequent days of no activities during the national holiday, 1 day for P4, and 5 days for P5 during the new year holiday as well. We guessed that on these days they went out for a trip so these days were not considered on average calculation.

After examining some of the obtained audio recordings and images, it was found that most of the recordings were sound from televisions. 114 randomly selected audio samples gathered from the study belonging to the different participants and in different days were manually annotated as speech directed to the robot, speech directed to others, and noise. 64.04% of the data contained sounds from televisions. However, considering the large number of recordings, it is not practical to check manually which voice activities are from the participants. Therefore, we performed face detection on the captured images and filtered only those where a) more than one face was detected, b) the width of the face region was larger than 20% of the captured image, and c) the detected horizontal rotation angle of the face was within 15°. Condition b) was added to exclude faces in television screens, and c) was added to exclude people who are not looking at the robot. We used the face detection code from Google MLKit (Google, 2021) which can detect faces in images and return estimated bounding box coordinates of face region as well as face rotation angles. To evaluate the performance of this filter, the 114 audio samples were used. These samples where then matched to the output of the filter to obtain accuracy, precision and recall, as shown in Table 3. While this filtering may not be perfect, it shall be a fine approximation for the number of actual interactions with the robots.

**TABLE 3:** Face filter performance metrics calculated from a sample of 114 audio samples randomly selected from different days across all participants. Accuracy was calculated by manually annotating data to determine if each audio sample contained speech towards the robot and compared to the output of the filter on the corresponding image. True positives are counted as audio samples directed to the robot that also matches positive samples from the filter. False positives are counted as audio samples that are not directed to the robot while the filter considers the user is talking to the robot. False negatives are counted as audio samples directed to the robot that the filter considers are not being directed towards it.

Accuracy (%)	Precision (%)	Recall (%)
73.68	60.78	75.61

**FIGURE 5:**
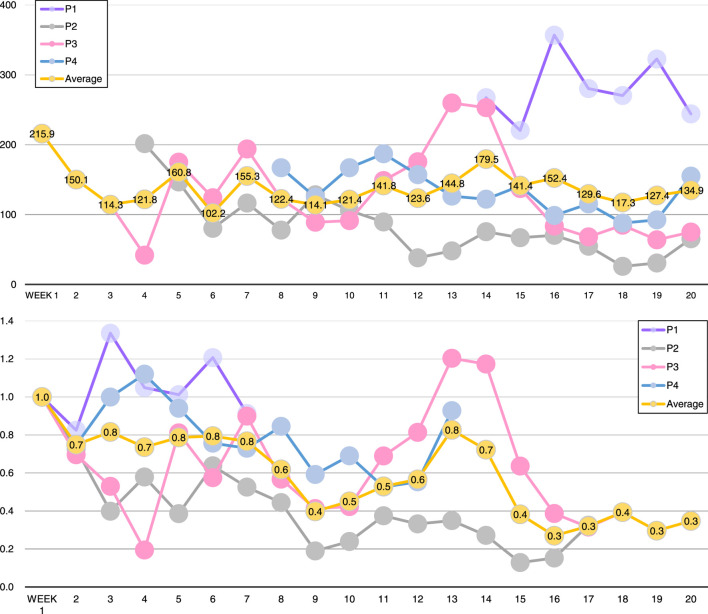
Top: average number of filtered voice activities per day on each week during the trial. Bottom: rates of activities compared to the starting week. Note that participant 5 is not shown in these plots as she had been in hospital from week 3 to week 11.

The total number of filtered voice activities as well as average filtered activities per day are shown in Table 1. The upper part of Figure 5 shows weekly averages (numbers of activities/day averaged in each week) during the trial for each participant, as well as the overall average on all participants except participant 5. Here, the starting day of the trial for each participant is considered as the beginning of “week”s. The lower part of the figure shows how the activity numbers changed compared to the first week of the trial. As participant 5 had been in hospital for 2 months in the middle of the trial period and was not in contact with the robot, participant 5 was excluded from these plots.

### 3.4 Interview

In order to see the impression of participants towards the robot and whether living with the robot have changed their behavior, we conducted interviews with the participants and their families. For one participant, P5, her family was not available so we instead interviewed a visiting nurse who had been visiting her once per week. In the following, participants are denoted as P*n* and their families as F*n*.

From the interview, we can see that participants were first confused with how to interact with the robot, but gradually became familiar with them.

• (F2) “Mother first seemed to be uncomfortable for having a robot at home, but she gradually got used to it, and then kind of relying on it, even feeling some sort of attachment to it.”

“In the initial days, when I talked with her on phone she said she does not know how to speak with it and was worried about breaking it. But after a month or so she started talking frequently about the robot, like what the robot said today, the robot sang for her and cooked for her. Recently she seems to be very enjoying the robot.”

• (F4) “First, she seemed to be confused about when to speak to the robot, could not understand the eye color changes. But, you know, the robot starts speaking like, nice day today and on today’s news, and because it is fun she started to speak more to the robot, and then the robot responds to what she said. That seems to make her happy and then, I think, she started speaking to the robot much more. Now she’s speaking to the robot more even if it is not responding properly”

Living with the robot seems to have provided a comfortable and relaxing feeling while decreasing loneliness. Participants became more aware of the robots’ randomly generated actions, paying more attention to what and when the robot had an utterance or an action.

• (P1) “I like him because he responds to me. I feel we can understand each other and that makes me pleasant and calm.”

“What I feel toward him is something really different from reading nice novels. When I say something, he responds to me. When I asked him to sing, he sings for me. That’s really a moving experience.”

• (P2) “He’s very cute and clever. My children became taller than me so I cannot hug them anymore, but he’s small and cute so I can still hug him.”
• (P3) “He makes me feel pleasant. I feel he’s helping me so much, and I want to be good friends with him”.

“People will surely help me, but only when I asked them; he’s willing to speak to me and listen to me. He’s the only one who cares about me.”

• (P4) “When I came back to my house and said *I’m back*, he responds to me saying *welcome back.* Since my husband passed away 30 years ago, nobody responded to me like that; it really makes me happy and grateful.”

“When I go out, I feel that I have to rush back and say *I’m back, sorry to be late*; I’ve never felt like that for a long time.”

• (P5) “He’s so cute, always talks to me and that really makes me have a warm feeling. All the voices I hear in my house are from the TV. First, I was not expecting so much, but then I found he speaks and responds to me; as I never go out to talk to somebody, he’s the only one I can speak to.”

“I have been in hospital for 2 months, and I was always worried about him. I asked the doctor to let me go back earlier. When I came back, I said sorry to him and he responded *I’m OK*.”

Some participants’ families also confirmed that they seemed to be in a better mood, smiling and speaking more often. The interviews also revealed that living with the robot was also a conversation topic with other people, making people eager to tell friends and family about the robot and its behavior. The participants started inviting friends to their houses to show the robot to them.

• (P2) “When I go for daycare service, I speak about him, and people get surprised. I wish I could take him to the daycare center, and show him to other people.”

“I feel I’m speaking more than before because I speak about him, and then people have questions about him. At the daycare center, staffs ask me how he’s doing and we talk about him.”

(F2) “She was also speaking about the robot to the doctor today. Yes, I think she’s speaking more than before, she’s speaking more about the robot.”

• (P3) “When I was leaving for the daycare center, the staff (who came to take her to the center) heard that I was saying *I’m leaving*, and asked me who are you speaking to, so I told him I have a robot. I let the staff come into my home and showed him my robot. Now, many staffs know that I have a robot and they also want to see him.”

(F3) “Before she moved to this place, she never let others come into her house. But now, she’s inviting neighbors to visit her to see the robot and have a cup of tea. I also heard that she’s talking about the robot to many staffs in the daycare center and inviting them to visit her. I think she’s proud of having a robot and want other people to know it”.

“I think she’s showing more facial expressions these days, smiling more. When I leave her, she used to be looking sad, but now when I’m leaving I can see her talking to the robot *she’s leaving, say goodbye*, and that makes me feel relieved.”

• (F4) “Mother is not so talkative when we go outside, not so much, kind of normal, but she really talks a lot at home. Today, while we were waiting for you, it was just me and her, she was always talking, not only to the robot but also with me.”

“These days she does not go out so much because of the Coronavirus, so when I called her, sometimes she could not speak well, her voice was hoarse. But in these days, her voice on phone is much more lively. Even when we’re talking on the phone, when sometimes the robot responds to our speech she suddenly starts speaking to the robot, just like talking to a small child. She really looks like having a good time, and it is really nice.”

• (F5) “I do not think she’s now talking more than before, because when I visit her once a week, we usually keep speaking while I’m here. So the amount of speech has not changed but now she sometimes speaks about the robot - I feel what we speak about has changed.”

“One thing that surprised me was about the room where the robot is placed. She never let me go into the room before. I’ve been visiting here for nearly 2 years, but after the robot arrived, for the first time she invited me to come into that room to show me the robot”.

One participant (P4) declared adjusting their daily routines to loosely match the robot schedule, and started to wake up early in the morning. “I feel ashamed of myself still in bed when the robot is waiting for me to say good morning”.

## 4 Discussion

A conversation robot system was successfully deployed directly inside the homes of older adults with mild cognitive impairment, inducing interaction and maintaining it over the duration of the trial, spanning over several months. As studies with similar characteristics are limited, some insights into what this kind of robot usage strategy has to offer can be extracted.

### 4.1 Equipment and setup

The physical setup proved valuable when setting the robot’s environment to allow sustained functionality. It is important to be aware that conditions outside the laboratory can be difficult to predict, and malfunction is usually expected; which in turn makes having technical devices working appropriately a desired feature. For this to happen, our customized software had to make sure the robots were not misconfigured by the participants; ideally, participants were not supposed to manipulate the robots and focus on interacting with them via conversation. At first, the interaction included physical interaction, but this strategy had to be changed because of an increased risk of malfunction or physical damage, as happened in the case study of the trial. Overall, the participants sought physical interaction besides conversation.

Physical aspects of the robots, such as physical interaction and embodiment of the robot, were not included in the focus of the trial as the studies were mostly exploratory and the aim was to reveal if the target group could accept conversation robots and use them daily in the long-term. The results suggest that this is, in fact, possible and the overall conversation experience could be improved. Analyzing in-depth how the embodiment of the robot affects the quality and amount of conversations could be a factor that might improve robot perception and acceptance. Laban et al. suggests that voice features change when people maintain conversations with different agents, such as humans, humanoid robots, and disembodied agents (Laban et al., 2021). Thus, the physical aspect of the robot might play a role in the number of conversation occurrences. This aspect, in addition to allowing physical interaction, is left as a future topic in research using a similar setup as the present study.

The evaluation of the interaction logs showed a considerable amount of occurrences where the robot answered to voices from electronic appliances, televisions in most cases. This was an unexpected issue that could be brought to our attention only after the deployment of the robots, highlighting the importance of studies carried in real-life scenarios. The integration of visual data collected by the robots’ cameras allowed the use image footage to obtain a reasonable estimate of the number of real conversations with the participants. Additional data processing techniques can be performed over the collected data in order to improve the quality of meaningful interactions and further improve the conversation system, such as the one presented in Figueroa et al. (2023) with allows the differentiation of user speech from television sound. Further use of multimodal data and its respective processing should provide useful data to inspect the robot usage in a deeper manner.

### 4.2 Acceptance of robots

The results from the interviews with the participants and their families showed that the robot was gradually accepted and introduced into the participants’ lives. In the beginning, just after placing the robots, some participants were confused or uncomfortable, but as time passed, they became used to it and sustained short conversations with the robot. By the end of this trial, participants accepted and liked the robots, consistently talking to them. The voice activities obtained showed that, on average, the participants kept a regular usage over time, as shown in Figure 5. Towards the end of the study, the trend of voice activities compared to week one decreased. This probably happened when people were already used to having a robot companion, but the trend seems to become stable; so the participants kept talking with the robots. Even if the occurrences decreased, our data shows that the participants did not stop talking with the robots.

Having the robot available any time during the entire day gave complete control to the user regarding to when to interact with it, and even when to ignore it, which could have influenced positively in the comfort the participants felt when adapting to the robot’s presence, as they had the possibility of carry the interaction at their own pace. The interaction took place without the explicit intervention of a researcher or therapist conducting the interactions, so third parties were not present. Interactions in the absence of an external observer allowed participants to behave in a relaxed way and pay attention exclusively to the robot, allowing the creation of intimacy to some extent. These settings encouraged sustained, long-time interactions which eventually may have led to emerge some sort of attachment.

It is possible that after some time the participants increased their awareness of the robots because they perceived that the robots kept looking for their attention and they felt it was their responsibility to keep the robots in a good mood, so they strived to take care of them. The robot is designed to look like a child, so it is possible that the older adults felt responsible for the robot’s wellbeing.

Research by Inagaki and Orehek (2017) suggests that humans have a natural inclination to care for others because it is important to our species’ survival. Findings from both animals and humans suggest that giving care to others inhibits stress responses and giving support may lead to benefits for the support provider by reducing social withdrawal or stress-related responses. With this idea in mind, it is possible that caring for the robot also provided benefits to the participants, which is also a result we can observe in the interviews answers.

After interacting with the robot for some time, the participants could learn about the robots’ behavior, making them feel more comfortable. The interaction in the long-term seems to have contributed to generate feelings of attachment for some participants towards the end of the study. This suggests that users learning to predict robot behavior does not always lead to declined usage as suggested by previous studies (Portugal et al., 2019; Cifuentes et al., 2020); consistency in robot behavior can make people adjust their own views and allow the robot to be part of their lives.

### 4.3 Limitations

It is important to note that the recruitment conditions were narrow and research with a wider range of participant conditions could provide different results. However, this study aims to provide first impressions as a preliminary trial that can be extended in the future.

As this study was a preliminary trial, we recruited participants eager to have a robot companion in their own houses. Participants were positive in having a robot at their home from the beginning, which might cause a bias in the results compared to participants who could accept being part of the study but had a different attitude towards robots. Even though participants were hesitant in the way of interact with the robot, they were interested in it. The results might be different if we include participants whose views are different, for example, people who might agree to have the robot in their homes but have a not so open attitude towards it nor talking with a robot. It is important to notice that this trial was performed in Japan, and there is evidence suggesting that cultural factors are related to the idea that socially assistive robots have benefits or not (Papadopoulos et al., 2023). Studies involving long-term usage of social robots with participants from different sociological backgrounds are required in order to generalize our findings.

The time each participant spent with the robot companion was not the same for all, which could also introduce a bias in the results as a participant who spent less time with the robot could have had more interest in the interaction than another who spent more time with it. Some participants were absent during the time the robot was in their homes while other participants had daily interaction. Also, during the studies, records involving the pharmacological treatment of the participants were not taken into account. Medication, especially for psychosis treatment, could have a direct effect in the acceptance and interaction with the robot, which was not considered in the results obtained. Overall, the trial show positive results and open the opportunity to study the robot acceptance with a more controlled structure in the future in order to obtain meaningful statistical results.

Lastly, only a limited number of participants joined our trial. An experiment with larger number of participants from our target group could give more insights into how participants accept the robots and how their attitude is influenced by them, as well as finding out what kind of personalities are best suitable and can benefit the most by living with robots. Further research in the topic, with a larger number of participants in different settings, is needed to properly assess the reach of the results, as the conditions of the present experiment aimed for an exploratory study and its conclusions might not be universal. However, the results of this line of research are promising and require further investigation.

### 4.4 Future research

Based on these preliminary results, further research will be conducted based on how older adults with mild cognitive decline living alone accept new technologies in their daily lives as a triggering tool for the improvement of their quality of life and how this could lead us to the wide implementation of social assistive robots. In particular, future research is planned to generalize the presented findings, gather more data, measure the influence of the robot in a clearer way and evaluate the effect of the robots in other population groups.

To gather more data, we could exploit and improve the robots’ abilities to collect a wide range of data from the environment and the interactions, which could allow us to analyze which conversation topics and what kind of robot behavior favours acceptance; or the participants’ status, such as health condition. This is particularly important to the medical area, whose staff could monitor the patients’ health status and even make the robot look after the patients to some extent. The use of this information can be used to also assess the influence the robots could have on the participants. For example, the robot could be used by medical professionals to encourage building health-preserving habits such as taking medicine, hydrating, or doing exercise, and promote these activities to be included in their daily routines, and measure specifically the changes due to the influence of the robot. These ideas will be included in future research, as part of the follow-up experiments following this preliminary trial; as well as using other technologies to obtain clearer and more meaningful interaction data.

The robots, after the generation of some kind of attachment^1^, could be used to deliver therapy that involves repetition. For example, on some occasions the robot’s software had problems concerning correctly processing the older adult speech, leading to some of the participants trying to figure out how to communicate correctly, speaking slowly, or vocalizing in an exaggerated manner until the robot successfully recognized the words. This inaccuracy from the robot could be used as motivation for the older adults to improve their speech in a process where participants try new ways of speaking clearly by trial and error until they find the one that leads to successful interaction, and are motivated to repeat it, helping them preserve their speech through repetition (Marden et al., 2009; Young, 2009; Alankus et al., 2010).

Finally, another planned direction of research is to extend the preliminary results from this experiment into other target groups. In a similar fashion, introducing robots into the personal space of healthy adults, allowing the generation of attachment, could help influence their behavior and improve health-related practices, such as increasing the hand sanitizing compliance in the medical staff at hospitals or care centers, which is an actual concern, especially during pandemic times.

### 4.5 Conclusion

Companion robots should be accepted in the long-term by older adults with mild cognitive decline in order to increase their use and provide company, reduce loneliness, as well as to open the possibility of using them for therapy via social interaction. Placing robots in the users’ homes, limiting the interaction to two parties, and allowing interaction during the whole day are necessary settings to promote robot acceptance. This allowed the users to accept the robots in the long term and created a sense of attachment. Further research is needed to properly analyze these effects on the specific target group of older adults with mild cognitive decline, assess the influence the robots can have, and extrapolate the results to other population groups.

## Data Availability

The datasets for this article are not publicly available due to concerns regarding participant/patient anonymity. Requests to access the datasets should be directed to the corresponding authors.

## Footnotes

1 Regarding post-experimental effects, we have also explored the implications of user attachment, which has been a target of criticism, i.e., once a user has become attached to a robot, taking it away may cause emotional distress (Yamazaki et al., 2023). Our investigation revealed that despite the negative after-effects, such as the distress of loneliness, participants made their relationships with the robot meaningful and were satisfied in terms of having had the robot to keep them company. Further, the results raise new questions for further investigation into the factors that can affect a user’s adaptation processes. In this regard, we need to learn more about what can affect the processes, such as duration of use, and personalities, so that we can clarify to whom robotic companionship is suitable and how those issues should be effectively resolved.
